# Effect of seawater salinity, pH, and temperature on external corrosion behavior and microhardness of offshore oil and gas pipeline: RSM modelling and optimization

**DOI:** 10.1038/s41598-024-67463-2

**Published:** 2024-07-17

**Authors:** Imran Mir Chohan, Azlan Ahmad, Nabihah Sallih, Naraindas Bheel, Waleligne Molla Salilew, Abdulrazak H. Almaliki

**Affiliations:** 1 Department of Mechanical Engineering, Universiti Teknologi PETRONAS, Tronoh, Bandar, 32610 Seri Iskandar, Perak Malaysia; 2 Department of Civil and Environmental Engineering, Universiti Teknologi PETRONAS, Tronoh, Bandar, 32610 Seri Iskandar, Perak Malaysia; 3 Mechanical Engineering Department, Addis Ababa Science and Technology University, Addis Ababa, Ethiopia; 4 Department of Civil Engineering, College of Engineering, Taif University, 21944 Taif, Saudi Arabia

**Keywords:** Offshore pipeline, Carbon steel pipe, Pipeline external corrosion, Marine water, Environmental sciences, Ocean sciences, Materials science

## Abstract

This research aims to investigate the effects of seawater parameters like salinity, pH, and temperature on the external corrosion behaviour and microhardness of offshore oil and gas carbon steel pipes. The immersion tests were performed for 28 days following ASTM G-1 standards, simulating controlled artificial marine environments with varying pH levels, salinities, and temperatures. Besides, Field emission scanning electron microscopy (FESEM) analysis is performed to study the corrosion morphology. Additionally, a Vickers microhardness tester was used for microhardness analysis. The results revealed that an increase in salinity from 33.18 to 61.10 ppt can reduce the corrosion rate by 28%. In contrast, variations in seawater pH have a significant effect on corrosion rate, with a pH decrease from 8.50 to 7 causing a 42.54% increase in corrosion rate. However, the temperature of seawater was found to be the most prominent parameter, resulting in a 76.13% increase in corrosion rate and a 10.99% reduction in the microhardness of offshore pipelines. Moreover, the response surface methodology (RSM) modelling is used to determine the optimal seawater parameters for carbon steel pipes. Furthermore, the desirability factor for these parameters was 0.999, and the experimental validation displays a good agreement with predicted model values, with around 4.65% error for corrosion rate and 1.36% error for microhardness.

## Introduction

Global oil and gas sectors depend on a vast network of carbon steel pipelines for producing and transporting hydrocarbons due to their distinctive properties like high strength, good ductility (yielding behaviour), low weight, cost effectiveness, weldability, and wear resistance^1^. The estimated length of those networks in 2017 was approximately 3.5 million kilometres, which is greater than nine times the distance between the Earth and the moon^2^, making them the world’s prime logistics infrastructure. Meanwhile, these megaprojects could not avoid failure problems, which were outnumbered by a variety of factors and causes. Among these, corrosion ranks as the second most common cause of metallic pipe failure^3^. Corrosion is the primary cause of metallic pipeline deterioration, damage, and failure, and it may also contribute to economic issues and negatively impact both humans and the environment^4^. Corrosion weakens the mechanical properties of pipes (hardness, toughness, ductility, and so on), increasing the probability of failure due to mechanical breakdowns^5,6^. Corrosion is also considered a crucial factor that influences the maintenance costs and lifespan of pipeline^7^.

Corrosion comes in a variety of forms^8,9^ and it is an electrochemical reaction between carbon steel and its internal and external environment^10^. Corrosion can cause internal and external metal loss in pipelines due to a variety of factors, including the properties of pipeline materials, environmental conditions, and the characteristics of the medium that flows inside the pipeline^11^. The characteristics of the medium, such as velocity, temperature, and pressure, commonly cause internal corrosion in pipelines^12^. On the other hand, the environment to which the pipeline is exposed influences external corrosion^13,14^.

Offshore marine pipelines that transport hydrocarbons in harsh sea environments are more prone to external corrosion due to the harsh conditions of seawater. Furthermore, there are many factors that contribute to the external corrosion of offshore pipelines, such as seawater microbiological influence, water alkalinity, seawater salinity, and seawater temperature^15,16^. The fluctuation in these sea parameters can substantially reduce the corrosion rate and enhance the pipeline's performance. Therefore, numerous scholarly studies have previously examined the impact of these seawater parameters on corrosion behaviour and pipeline material performance^17–19^. For instance, Wang Xinhua^20^ studied the corrosion behaviour of 2Cr13 stainless steel in different artificial seawater environments in 2020, considering pH, temperature, and dissolved oxygen contents. His study revealed that the temperature of seawater had a major impact on the corrosion rate, and a small 10 °C decrease in seawater temperature had an exponential impact on the corrosion rate. Furthermore, in 2020, Smith et al*.*^21^ conducted an immersion test to explore the impact of seawater salinity on mild steel with various chemical compositions. Their study demonstrated that increasing sodium chloride (NaCl) concentrations from 0.05 to 3.5% leads to an increase in corrosion rate, while increasing sodium chloride concentrations to 10% leads to a decrease in corrosion rate. Likewise, in 2019, Darmawan et al*.*^22^ investigated the influence of salinity on aluminium alloys in artificial seawater and reported a similar correlation. The reason for these finding could be a reduction in oxygen solubility accompanying increased water salinity, subsequently lowering water conductivity and, consequently, diminishing the corrosion rate^23,24^. Moreover, Chen et al.^25^ examined the corrosion behaviour and mechanical properties of low-alloy steel under different environmental conditions. Their study revealed a substantial decline in the mechanical properties (Ultimate tensile strength (UTS), Yield strength (YS), and elongation to failure (EFL)) of low-alloy steel due to corrosion in varying environments. Gao et al.^26^ investigated how saline water cathodic protection potentials altered the microhardness of Q235 steel using electrochemical impedance spectroscopy on steel samples. Their observations also indicated a decrease in microhardness over time. Despite the number of scholarly works presented in the literature about the effect of seawater parameters on corrosion inhibition of offshore pipelines, the combined impact of these parameters has not been well defined so far. Therefore, this research aims to fill that gap by studying the combined effect of these seawater parameters (salinity, pH, and temperature) on the corrosion behaviour and microhardness of oil and gas pipelines. Additionally, RSM modelling is applied to find out the optimal values of these parameters at which the oil and gas pipeline material has optimal performance.

## Materials and methods

**Figure 1:**
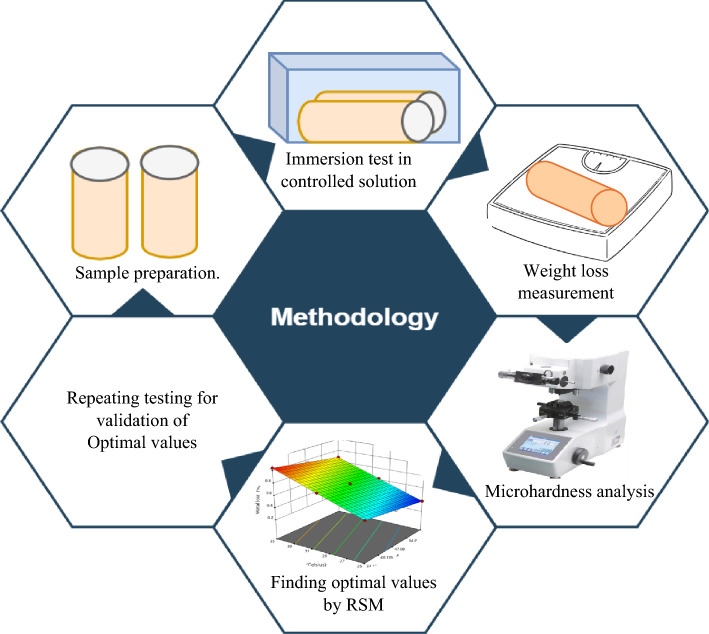
Methodological frame work.

The methodological framework used to investigate the effects of seawater parameters such as salinity, pH, and temperature on corrosion behaviour and microhardness can be seen in the Fig. 1. However, about materials and the concise techniques are provided in subparts of this section.

### Materials

ASTM 106 grade B carbon steel pipe having an inside diameter of 76.2 mm and an outside diameter of 88.9 mm is used in this study. The chemical composition and mechanical properties of pipeline are shown in Tables 1 and 2.

**Table 1:** The composition of carbon steel grade B pipe.

Element	C	Mn	P	S	Si	Cu	Ni	Cr	Mo	Fe
Quantity (%)	0.30	1.06	0.035	0.035	0.10	0.40	0.40	0.40	0.15	97.12

**Table 2:** Mechanical properties of ASTM 106 grade B carbon steel pipe.

Tensile strength (MPa)	Microhardness (HV)	Elongation (%)	Yield strength (MPa)
455	148	21	240

### Sample preparations

**Figure 2:**
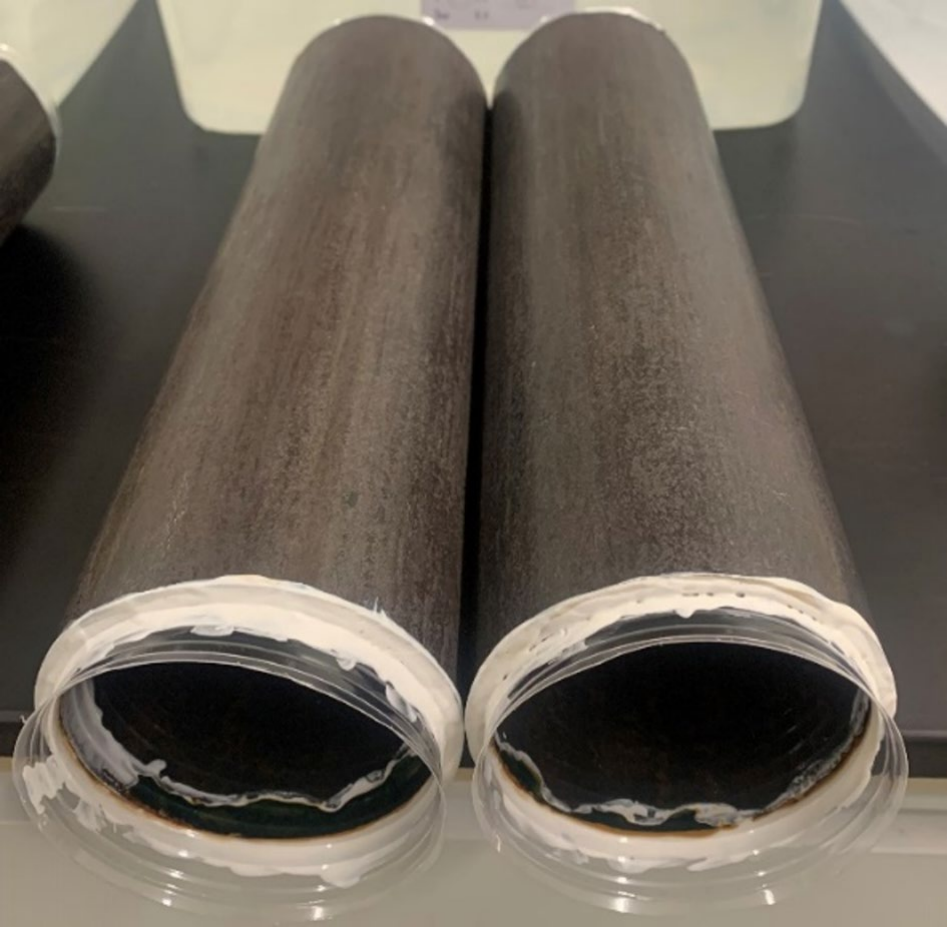
Pipe samples.

In this research, a pipe made of carbon steel with a wall thickness of 12.7 mm and a diameter of 3 inches is utilized. Using an abrasive wet-cutting machine, 1-foot-long samples of identical length were cut from each specimen. The external coating was then removed, capped with plastic caps, and sealed on both sides with a chemically resistant material to prevent leakage to the inner layer of the pipes and to guarantee that only the external surface area of the specimen is exposed to the artificial marine environment (see Fig. 2). Moreover, smaller coupon samples, measuring approximately 10 mm in length and 7.5 mm in width, were made for further analysis via FESEM characterization and microhardness evaluation. To prepare these coupons, a series of sandpapers ranging from 120 to 2400 grits were systematically employed using grinding and polishing machinery, ensuring a smooth surface.

### Immersion test

**Figure 3:**
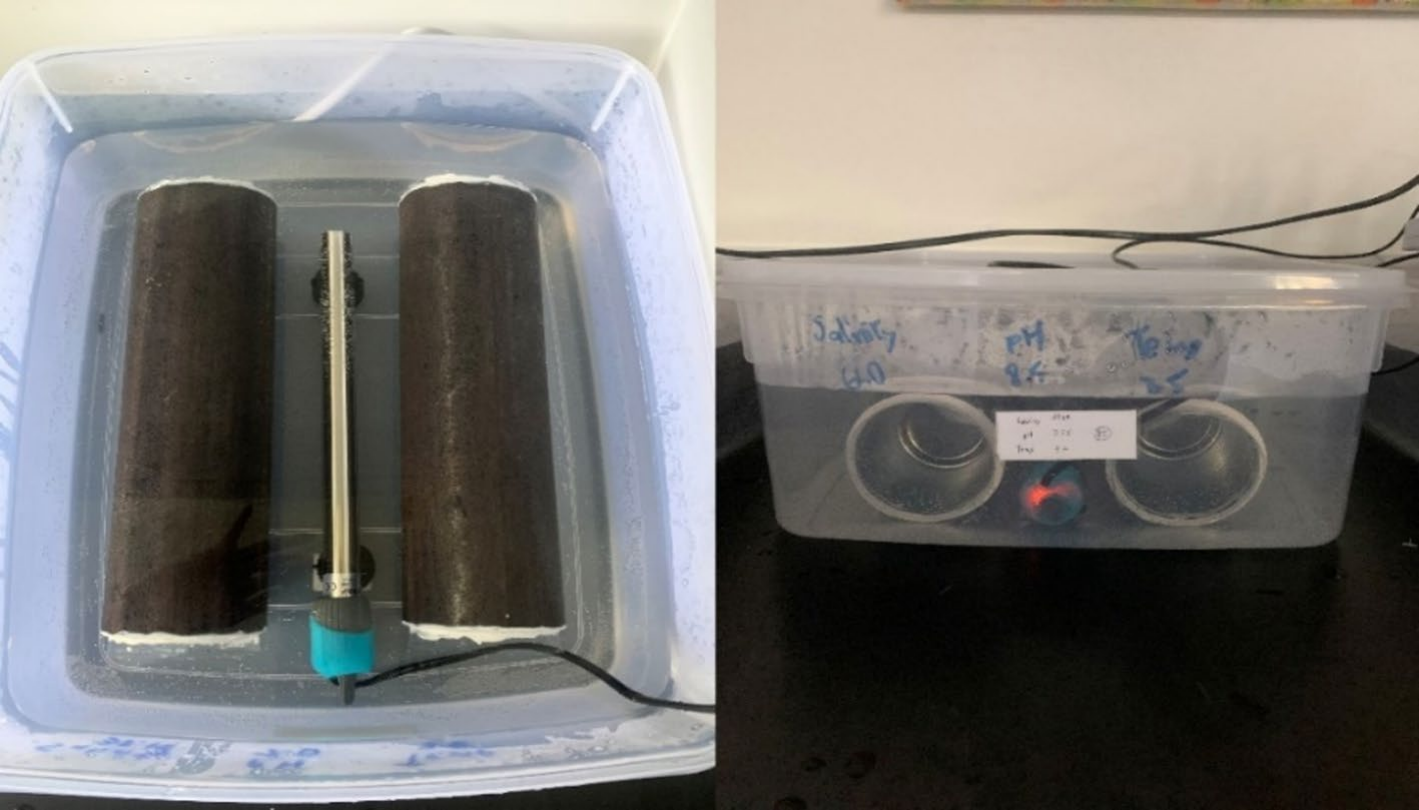
Experimental setup for immersion test.

In this study, a series of artificial seawater solutions with varying pH, salinity, and temperature were developed. Table 3 provides the ranges of the above-mentioned parameters and experimental conditions. These artificial environments replicate the conditions of the Malaka Strait and the South China Sea^27,28^. Furthermore these samples were immersed for 28 days following ASTM G-1 standards^29^. The temperature was controlled by electric temperature controller (see Fig. 3) and ranges were also selected for distinct seasons under identical marine conditions. In accordance with ASTM TM0169/G31 standards^30^, each test involved the exposure of two identical test samples along with coupons for FESEM and microhardness study. The immersion box was left open. Whereas by frequently adding the proper solution, the evaporation losses were maintained between + 1% and − 1% of the initial volume.

**Table 3:** Experimental parameter and ranges.

Parameter	Ranges
Temperature	25–35 °C
PH	7–8.5
Salinity	33.18–61.0 ppt
Exposure time	7, 14, 21 and 28 days

### Weight loss measurement and corrosion rate

After 28 days, the samples were taken out from the solutions and the rust was removed in accordance with ASTM G-31 standards^31^, so that only the corroded portion of the samples could be removed without affecting the pipe surface. The weight loss study of corrosion rate was conducted by comparing the pre- and post-exposure sample weights. Following the below equation, the rate of corrosion can be determined^31^.

1 $$Corrosion \; rate =\frac{(\text{K}\times \text{W})}{\text{A}\times \text{T}\times \text{D}}$$

where A = pipe exposure area, T = immersion duration in hours, K is constant = 87,600, D = density of sample that is 7.85 g/cm^3^ and W = weight loss in grams.

### Microhardness measurement

After a 28-day exposure to various artificial marine environments, the microhardness of all samples was assessed following the standardized test method (ASTM E92-82) using a Vickers hardness tester^32^. This assessment utilized a 300-gf load with a 25-s dwell time. Throughout the hardness test conducted at room temperature (25 °C), measurements were taken four times at different locations on each test sample, and the mean values derived from these measurements were used to determine the microhardness. Notably, the experimental error during the hardness assessment remained below 5%.

### Design of experiments developed by RSM modelling

The research employed the central composite design (CCD) approach within the Response Surface Methodology (RSM) to explore the impact of various factors and their interactions—sea water pH, salinity, and temperature—on both the corrosion rate and the microhardness of carbon steel pipes. This method allowed for the efficient development of a second-order model, crucial for understanding the variables' effects comprehensively. The CCD design is better for estimating control parameters in a second-order model because it includes more center and axial points, which make it more stable. The study utilized seawater salinity, pH, and temperature as input parameters, with Table 3 outlining their levels across the experimental process. The output parameters, corrosion rate, and microhardness, detailed in Table 4, were the focal response variables analyzed. The design matrix, which shows the factors in the surface response system in specific units, was carefully recorded in each testing run (R1–R17) and shown in Table 4. It also includes observations on the rate of corrosion and the microhardness responses.

**Table 4:** Design matrix (CCD).

Testing runs	Input parameters			Output responses	
	Factor 1 PH	Factor 2 Temperature (°C)	Factor 3 Salinity (PPT)	Response 1 Corrosion rate (mm/year)	Response 2 Microhardness (HV)
R1	7.75	30	61	0.292	140
R2	8.5	25	33.18	0.185	143.7
R3	7	35	61	0.516	134.9
R4	7.75	30	33.18	0.390	138.5
R5	7	30	47.09	0.419	137.9
R6	7.75	35	47.09	0.487	135.6
R7	8.5	30	47.09	0.282	140.5
R8	7.75	30	47.09	0.341	139.3
R9	7.75	25	47.09	0.195	143.3
R10	7.75	30	47.09	0.341	139.3
R11	8.5	35	33.18	0.477	136
R12	7	25	33.18	0.321	141.1
R13	8.5	25	61	0.0877	145.2
R14	7	25	61	0.224	142.6
R15	8.5	35	61	0.380	137.5
R16	7.75	30	47.09	0.341	139.3
R17	7	35	33.18	0.614	133.4

## Results and discussions

### Effect of marine water salinity on oil and gas carbon steel ASTM 106 grade B pipe

**Figure 4:**
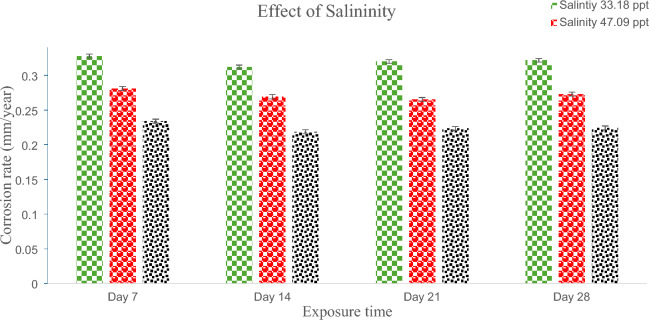
Effect of marine water salinity on CS pipe corrosion rate.

As previously indicated, the assessment of pipe sample corrosion when exposed to distinct salinity ranges within marine water solutions was conducted employing the weight loss method. The corrosion rate findings concerning offshore oil and gas marine pipelines subjected to varying timeframes of exposure and salinity are presented in Fig. 4. The corrosion behavior analysis for these pipelines took place at intervals of 7, 14, 21, and 28 days. Figure 4 provides a visualization of the external corrosion rate of a pipe exposed to seawater with a salinity concentration of 33.18 ppt. The external corrosion rates measured on the seventh, fourteenth, twenty-first, and twenty-eighth days were approximately 0.327685 mm/year, 0.312081 mm/year, 0.319883 mm/year, and 0.321833 mm/year, respectively. Notably, there is a decrease in corrosion rate from the 7th to the 21st day, followed by a subsequent increase on the 28th day. This trend was consistent across experiments conducted with different salinity concentrations, such as 47.09 ppt and 61.0 ppt, as illustrated in Fig. 4. The oxide layer which grows spontaneously on metal surfaces is mostly responsible for this phenomenon. This coating protects the metal from atmospheric corrosion by acting as a powerful barrier. However, over time, this oxide layer gradually decreases, and the corrosion rate begins to rise^33^. This observation parallels the findings of Royani et al., who observed a similar pattern in their investigation of the internal corrosion behavior of CS pipes within a freshwater environment^34^.

Furthermore, the impact of marine water salinity on the rate of external corrosion is pronounced. Figure 4 shows the external corrosion rates of CS pipes over 28 days for salinities of 33.18 ppt, 47.09 ppt, and 61.0 ppt, which are about 0.321833 mm/year, 0.273071 mm/year, and 0.224308 mm/year, respectively. The experimental results suggest that reduced water salinity enhances the external corrosion behavior of CS pipes. When the salinity goes from 33.18 to 47.09 ppt, the rate of external corrosion goes down by about 14.66%. When using CS pipes in marine environments with a salinity of 61.0 ppt, the rate of corrosion goes down by about 28%. These findings align with previous literature. For instance, in 2020, Smith et al.^21^ conducted an immersion test to explore the impact of seawater salinity on mild steel with various chemical compositions. Their study demonstrated that escalating NaCl concentrations from 3.5 to 10% led to a decrease in corrosion rate. Likewise, in 2019, Darmawan, Agung Setyo, et al.^22^ investigated the influence of salinity on aluminum alloy in artificial seawater and reported a similar correlation. This behavior can be attributed to the reduction in oxygen solubility accompanying increased water salinity, subsequently lowering water conductivity and, consequently, diminishing the corrosion rate^23,24^.

### Effect of marine water pH on oil and gas carbon steel ASTM 106 grade B pipe

**Figure 5:**
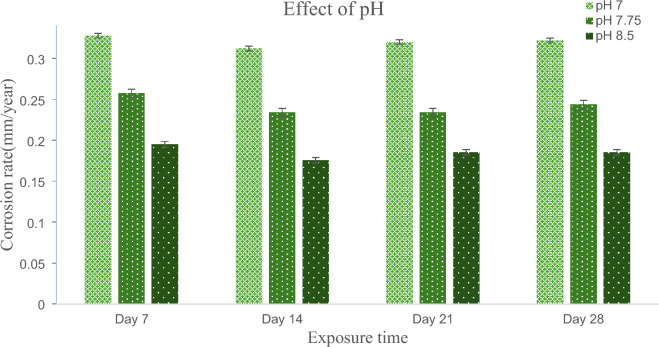
Effect of marine water pH on CS pipe corrosion rate.

The pH of Marin water has a major impact on the external corrosion behavior of CS pipe. According to Fig. 5, the corrosion rate of carbon steel pipes after 28 days in marine environments with pH 7.0, 7.75, and 8.5 is approximately 0.321833 mm/year, 0.243813 mm/year, and 0.185298 mm/year, respectively. The external corrosion behavior of CS pipe is increased when used in marine environments with a decreasing pH from 8.5 to 7.0. According to the results of the experiment, reducing the pH of seawater from 8.50 to 7.75 may increase the corrosion rate by 24.19%, and further lowering the pH to 7 may increase the corrosion rate by 42.54%. In 2015 similar results were observed by Pessu et al. where they performed the experimental study to find out the effect of pH on corrosion behavior of X65 carbon steel in CO_2_-Saturated brines^35^. Consequently the same agreement was noticed by Toloei et al.^36^ when they were conducting experimental work to study the effect of sea water pH on corrosion behavior of AISI 1045 carbon steel in turbulent condition. It has been observed that as the pH of the marine environment changes, the corrosion rates of carbon steel pipelines exhibit a discernible trend. As pH increases from 7 to 8.5, the carbon steel pipe's corrosion rate decreases steadily. This trend is consistent with the fundamental principles of corrosion chemistry, according to which an increase in pH generally reduces the corrosion rate of metals^37^. This is due to the formation of a passive film on the surface of the metal under higher pH conditions^38^.

### Effect of marine water temperature on Oil and gas carbon steel ASTM 106 grade B pipe

**Figure 6:**
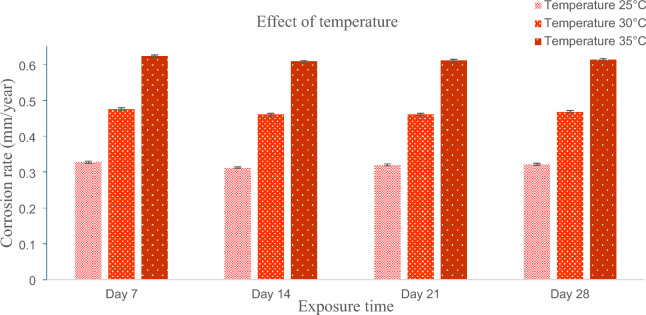
Effect of marine water temperature on CS pipe corrosion rate.

Based on the data provided in Table 3, the experiment involved maintaining a pH level of 7 and a salinity of 33.18 ppt while varying the temperature settings to 25 °C, 30 °C, and 35 °C. Throughout the immersion-based study, minimal fluctuations in the corrosion rate were observed, particularly on the 7th, 14th, 21st, and 28th days. The investigation focused on the corrosion rate of carbon steel (CS) pipes exposed to the environment over a 28-day period. The corrosion rates recorded were approximately 0.321833 mm/year, 0.468121 mm/year, and 0.614409 mm/year for temperatures of 25 °C, 30 °C, and 35 °C, respectively, as illustrated in Fig. 6. The results indicate a significant correlation between the temperature of the marine water and the rate of corrosion in CS pipes. Specifically, elevating the temperature from 25 to 30 °C resulted in a substantial 68.81% increase in the external corrosion rate. Furthermore, raising the temperature from 30 to 35 °C led to a remarkable 76.13% rise in the corrosion rate. The results of this experiment are highly congruent with prior research. For instance, in 2022, Mobin et al. conducted an experimental study on the impact of various additives, temperatures, and immersion times on the corrosion behavior of mild steel. Their research demonstrated that a 10 degree centigrade increase in temperature exponentially increases the corrosion rate of mild steel^39^. Furthermore, in 2020 Abdeen et al.^40^ researched the effect of temperature on corrosion behavior of 304 L stainless steel and found increasing temperature has significant effect on corrosion behavior. They found 3 times an increase in corrosion rate by increasing 20-degree centigrade temperatures. These findings underscore a direct relationship: higher seawater temperatures correspond to accelerated corrosion rates. This connection aligns with the established understanding that elevated temperatures expedite corrosion processes. The heightened kinetic energy of molecules in warmer water accelerates the underlying chemical reactions that contribute to corrosion^41^. As a result, the surface of the carbon steel degrades more rapidly under these conditions^42^.

### Corrosion morphology of ASTM 106 grade B carbon steel pipe

**Figure 7:**
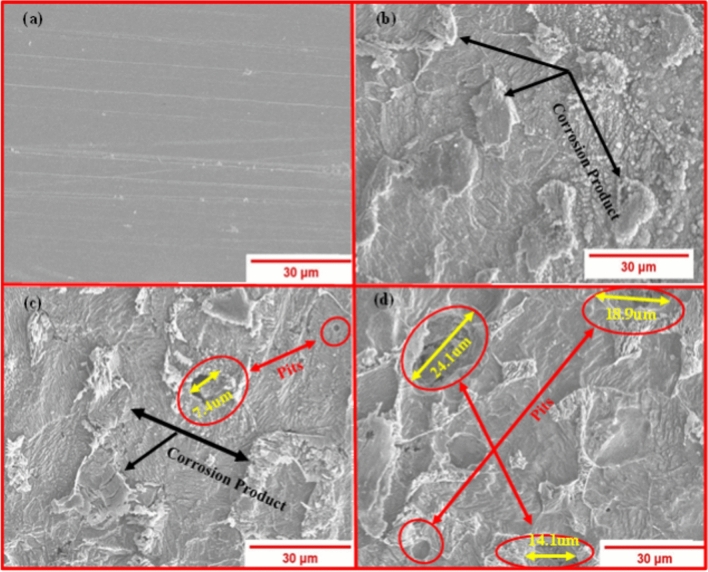
FESEM results of samples (**a**) before immersion test, (**b**) after 28 days immersion test with 25 °C temperature, (**b**) after 28 days immersion test with 30 °C temperature, (**c**) after 28 days immersion test with 35 °C temperature.

Since temperature is the most influential factor in the corrosion behavior of offshore oil and gas pipelines, there was a 76.13% increase in corrosion rate when the temperature was increased from 25 to 35 °C. Consequently, a FESEM examination was performed to examine the surface structure of samples to determine the type of corrosion. The FESEM images of pipe surfaces at 2000 × magnification before and after the 28-day immersion test in a controlled artificial marine environment with 25 °C, 30 °C, and 35 °C are shown in Fig. 7a–d, respectively. Evidently, before the immersion test, the sample surface was clean and free of any corrosion products, as shown in Fig. 7a. In Fig. 7b, the development of general corrosion was detected after 28 days of immersion testing in an artificial marine environment kept at 25 °C. The reason for this is the chloride ions that are present in the harsh, saline marine environment. The pipe's surface may become infected with general corrosion due to the extreme corrosiveness of these ions^43^. Furthermore, the FESEM image clearly indicates that no pit formation occurred in the sample within a 25-degree marine environment following a period of 28 days (see Fig. 7b). The formation of pits on the surface of the sample after a period of 20 days exposed to a controlled marine environment at a temperature of 30 °C is illustrated in Fig. 7c. A few little pits can be noticed, indicating a widespread occurrence of localized corrosion. The maximum width of the pit is approximately 7.5 µm. Besides Several pits were observed on the sample surface when it was exposed to the marine environment at 35 °C, as shown in Fig. 7d. It was observed that samples subjected to a marine water environment with a temperature of 35 °C exhibited larger pit sizes. The results presented in Fig. 7d indicate that the maximum pit size measured around 21.14 µm. This value is roughly three times larger than the pit size observed in samples subjected to a temperature of 30 °C. This observation highlights the substantial influence of marine water temperature on the development of pits on carbon steel pipeline surface. Consistent with the experimental results, the surface examination of samples subjected to different temperatures of marine water confirms the significant impact that temperature has on the corrosion properties of carbon steel (CS) pipes. Likewise, relevant observations have been recorded in extant scholarly works. In 2017, for instance, Okonkwo et al.^44^ conducted research to determine how temperature affected the corrosion behavior of API X120. A significant correlation was discovered during their investigation between water temperature and pitting corrosion in API X120 pipelines. An increase in water temperature from 20 to 40 °C was found to result in a noticeable widening of pitting corrosion on the pipe samples' surfaces^44^. It is worth mentioning that significantly increasing the temperature accelerates the rate of corrosion.

### Microhardness of carbon steel pipes

**Figure 8:**
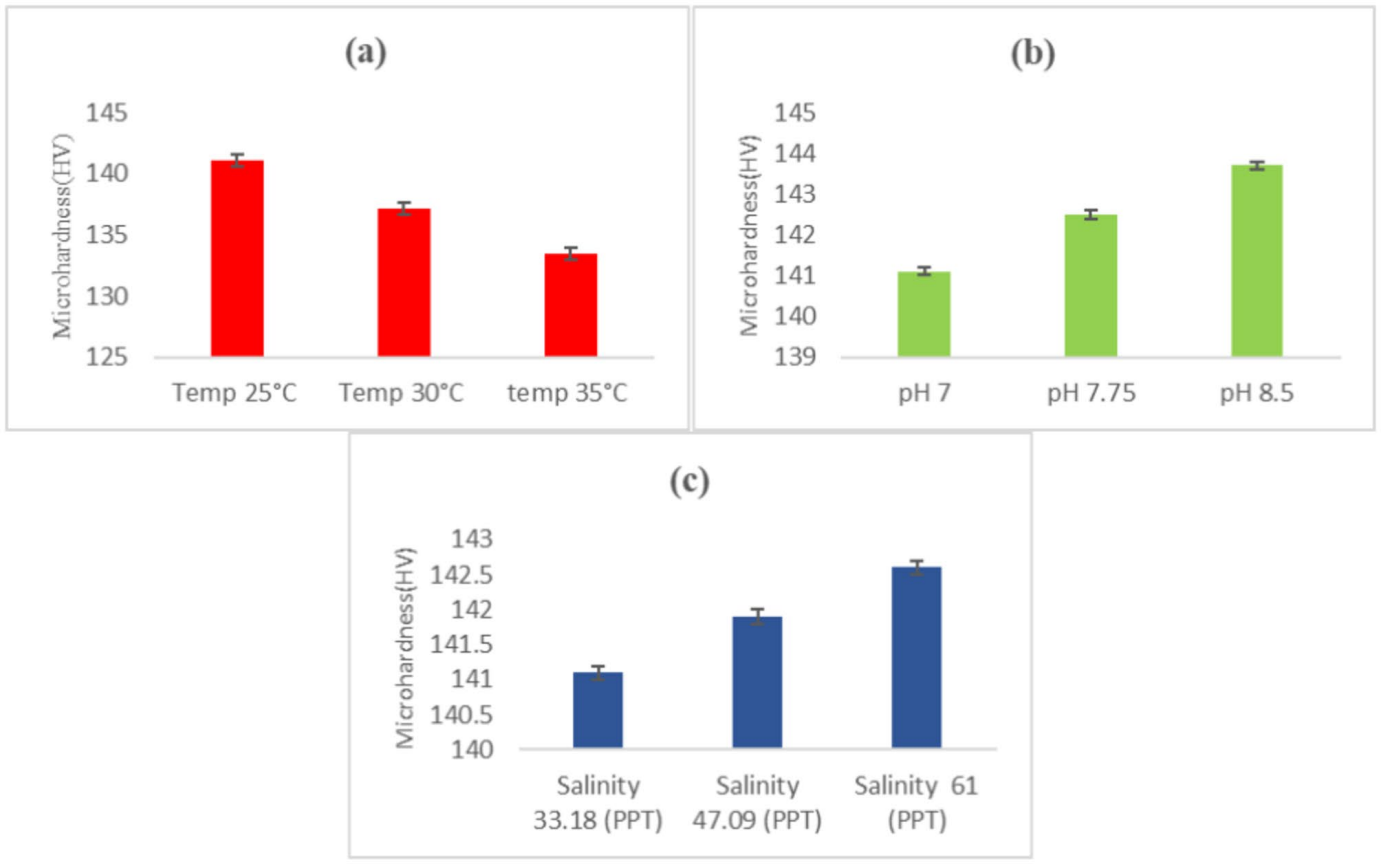
Effect on microhardness (**a**) sea water temperature, (**b**) sea water pH, and (**c**) sea water salinity after 28 days.

**Figure 9:**
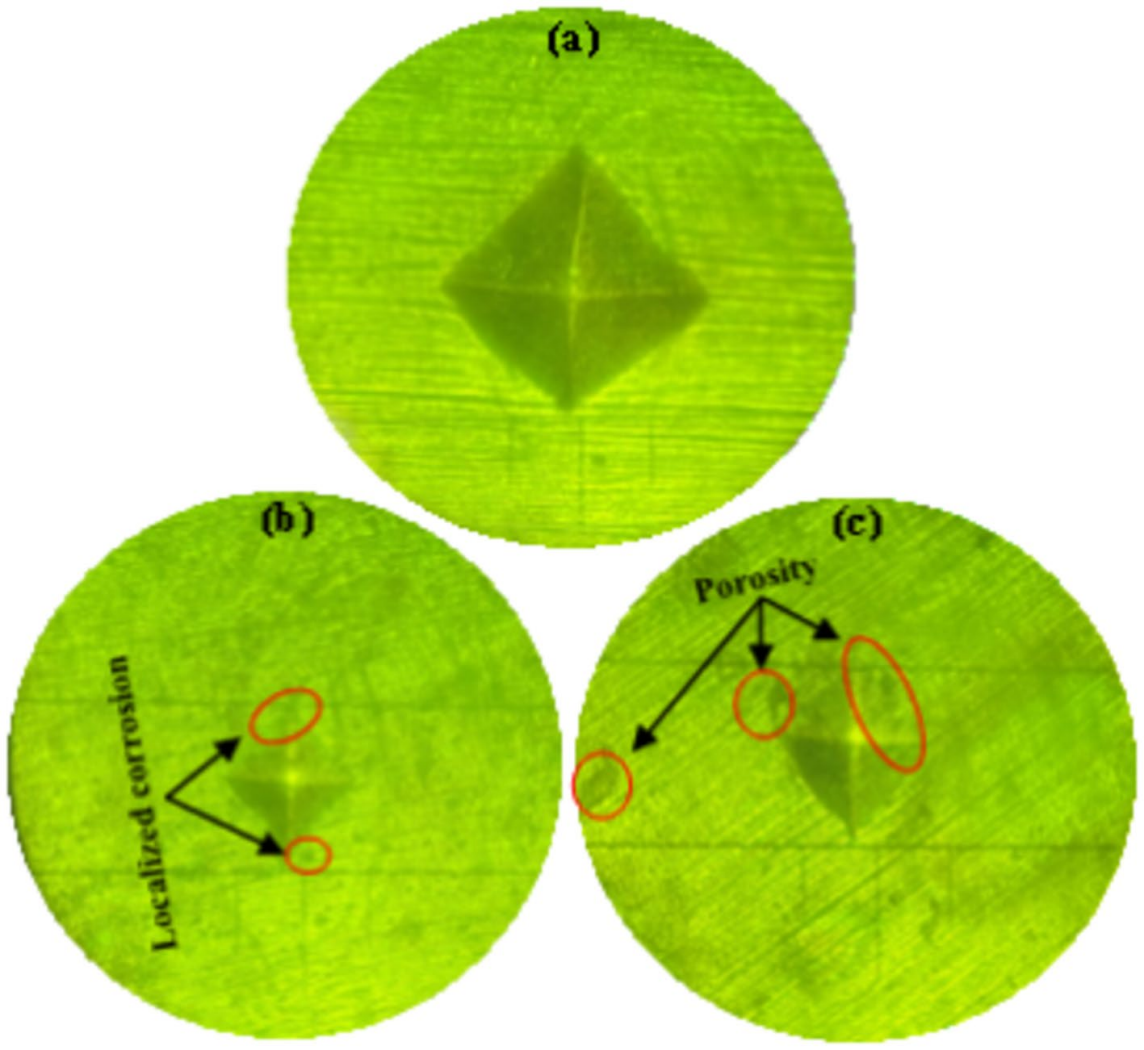
Image for microhardness indentation (**a**) fresh, (**b**) 30 °C, and (**c**) 35 °C after 28 days.

The surface analysis identified two types of corrosion, general and pitting, on carbon steel samples. To delve deeper, we utilized the Vickers tester to evaluate the microhardness of these samples under various environmental conditions over a 28-day period. Each sample underwent four microhardness measurements, and the average values are depicted in Fig. 8. The microhardness of carbon steel samples that were put in artificial marine environments at 25 °C, 30 °C, and 35 °C was 141.1 HV, 137.1 HV, and 133.4 HV, as shown in Fig. 8a. Figure 8b also shows that specimens that were put in controlled environments with pH levels of 7, 7.75, and 8.5 had microhardness values of 141.1 HV, 142.5 HV, and 143.7 HV, respectively. These samples were also put in seawater with salinities of 33.18 PPT, 47.09 PPT, and 61.0 PPT. After 28 days, they had microhardness values of 141.1 HV, 141.9 HV, and 142.6 HV (Fig. 8c). The experimental results highlight that seawater temperature has a considerably more pronounced impact—about 3 to 4 times greater—on the microhardness of carbon steel pipes compared to seawater salinity and pH. Notably, a mere 10 °C increase in marine temperature from 25 to 35 °C leads to a significant 10.99% decrease in the microhardness of carbon steel pipes. This reduction is attributed to prolonged exposure to seawater at specific temperatures, inducing material degradation that affects the surface microhardness of the samples^45^. Moreover, the pits that formed on the sample surfaces after 28 days of seawater exposure primarily contributed to the decrease in microhardness (refer to Fig. 9). Previous literature supports findings that are similar. For instance, Chen et al.^25^ examined the corrosion behavior and mechanical properties of low-alloy steel under different environmental conditions. Their study revealed a substantial decline in the mechanical properties (UTS, YS, and EFL) of low-alloy steel due to corrosion in varying environments. Gao et al.^26^ used electrochemical impedance spectroscopy on Q235 steel samples to find out how saline water cathodic protection potentials changed the microhardness of Q235 steel. Their observations also indicated a decrease in microhardness over time.

### RSM modelling

Using Design Expert software version 12.0, experimental data was incorporated into the development of the corrosion rate model, which is a comprehensive quadratic model with interaction, squared, and linear terms. Meanwhile, the microhardness model was constructed using a linear equation. The subsequent equations, which are presented below, enable a comparison of the corrosion rate and microhardness responses with respect to the input variables A = salinity (PPT), B = pH, and C = temperature (Celsius) in the Design Expert software (see Table 5).

**Table 5:** Input variables and DOE limits.

Parameters	Description	Type	Units	Minimum	Maximum
A	Salinity	Numeric	PPT	33.18	61.0
B	pH	Numeric	–	7	8.5
C	Temperature	Numeric	Celsius	25 °C	35 °C

2 $$Corrosion \; rate= +0.34-0.049\text{A}-0.068\text{B}+0.15\text{C}+0.000\text{AB}+0.000\text{AC}-2.500\text{E}-007\text{BC}+3.953\text{E}-007{A}^{2}+9.752\text{E}-003{B}^{2}-9.459\text{E}-008{C}^{2}$$

3 $$Microhardness=+139.30+0.74\text{A}+1.30\text{B}-3.85\text{C}$$

Table 6 showcases the ANOVA analysis for the corrosion rate utilized in deriving Eq. (2). The model's F-value of 1109.64 indicates its significant relevance. This value suggests less than 0.01% likelihood of such a large F-value arising from random variations. The lack of fit F-value of 8.260E−010 indicates insignificance concerning the model's lack of fit relative to pure error, showing a 100% chance of occurrence due to noise. This non-significant lack of fit aligns with our goal of achieving a well-fitted model. Moreover, Table 7 presents the ANOVA results for microhardness, contributing to Eq. (3). Here, the model's F-value of 822.12 signifies its significant nature, with a mere 0.01% probability of arising from random fluctuations. Similar to the corrosion rate model, the lack of fit F-value of 0.026 indicates insignificance, with a 100% chance of occurrence due to noise. This lack of significance in the lack of fit aligns with our objective of creating a well-fitting model. These outcomes closely resemble findings from previous studies.

**Table 6:** ANOVA for corrosion rate.

Source	Sum of squares	df	Mean square	F-Value	p-value	
Model	0.29	9	0.032	1109.64	< 0.0001	Significant
A-Salinity	0.020	1	0.020	693.91	< 0.0001	
B-pH	0.047	1	0.047	1631.17	< 0.0001	
C-Temperature	0.21	1	0.210	7490.04	< 0.0001	
AB	0.000	1	0.000	0.000	1.000	
AC	0.000	1	0.000	0.000	1.000	
BC	5.000E−013	1	5.000E−013	1.750E−008	0.999	
A^2^	3.745E−013	1	3.745E−013	1.311E−008	0.999	
B^2^	2.427E−004	1	2.427E−004	8.49	0.022	
C^2^	2.287E−014	1	2.287E−014	8.005E−010	1.000	
Residual	2.000E−004	7	2.857E−005			
Lack of Fit	2.203E−013	4	5.507E−014	8.260E−010	1.000	Not significant
Pure Error	2.000E−004	3	6.667E−005			
Cor Total	0.29	16

**Table 7:** ANOVA for Microhardness.

Source	Sum of squares	df	Mean square	F-Value	p-Value	
Model	170.49	3	56.83	822.12	< 0.0001	Significant
A-Salinity	5.36	1	5.36	77.56	< 0.0001	
B-pH	16.90	1	16.90	244.49	< 0.0001	
C-Temperature	148.22	1	148.22	2144.33	< 0.0001	
Residual	0.90	13	0.069			
Lack of Fit	0.072	10	7.195E−003	0.026	1.000	Not significant
Pure Error	0.83	3	0.28			
Cor Total	171.38	16				

Response Methodology (RSM) was used to analyze and make sense of the data from the central composites design. The results are shown in Table 8 for corrosion rate and Table 9 for microhardness. The analysis was carried out utilizing Design Expert software (Version 12) as a key component of this study. The summary of the response model for both corrosion rate and microhardness are detailed in Tables 8 and 9, respectively. In assessing adequacy, the indicators—R^2^, adjusted R^2^, and predicted R^2^—present a cohesive picture for both responses. Regarding corrosion rate, these indicators collectively suggest a substantial relationship, with an observed predicted R^2^ of 0.9989 reasonably aligning with the adjusted R^2^ of 0.9984. The minor difference of 0.0005 between these values underscores the reliability of the relationship. Similarly, the indicators assessing microhardness reveal a strong correlation, where the predicted R^2^ of 0.9939 corresponds well with the adjusted R^2^ of 0.9935, demonstrating a robust relationship. The analysis of the variance outcome for both models indicates that significant model terms are the main effect of the three process parameters (salinity, pH, and temperature of sea water) along with the interaction effect of the three parameters^24^.

**Table 8:** Model summary for corrosion rate.

Source	Std Dev	R^2^	Adjusted R^2^	Predicted R^2^	Press	
Linear	6.669E−003	0.998	0.997	0.997	8.549E−004	
2FI	7.604E−003	0.998	0.996	0.991	2.303E−003	
**Quadratic**	**5.345E−003**	**0.999**	**0.998**	**0.998**	**3.042E−004**	**Suggested**
Cubic	8.165E−003	0.999	0.996		+	Aliased

The Quadratic model recommended as the most viable option by the RSM tool (denoted by bold values among the four models listed in Table 8). Significant values are in bold.

**Table 9:** Model summary for microhardness.

Source	Std Dev	R^2^	Adjusted R^2^	Predicted R^2^	Press	
**Linear**	**0.26**	**0.994**	**0.993**	**0.993**	**1.04**	**Suggested**
2FI	0.30	0.994	0.991	0.993	1.05	
Quadratic	0.34	0.995	0.989	0.992	1.26	
Cubic	0.52	0.995	0.974		+	Aliased

The linear model recommended as the most viable option by the RSM tool (denoted by bold values among the four models listed in Table 9). Significant values are in bold.

### Effect analysis of seawater parameters on both responses (corrosion rate and microhardness)

**Figure 10:**
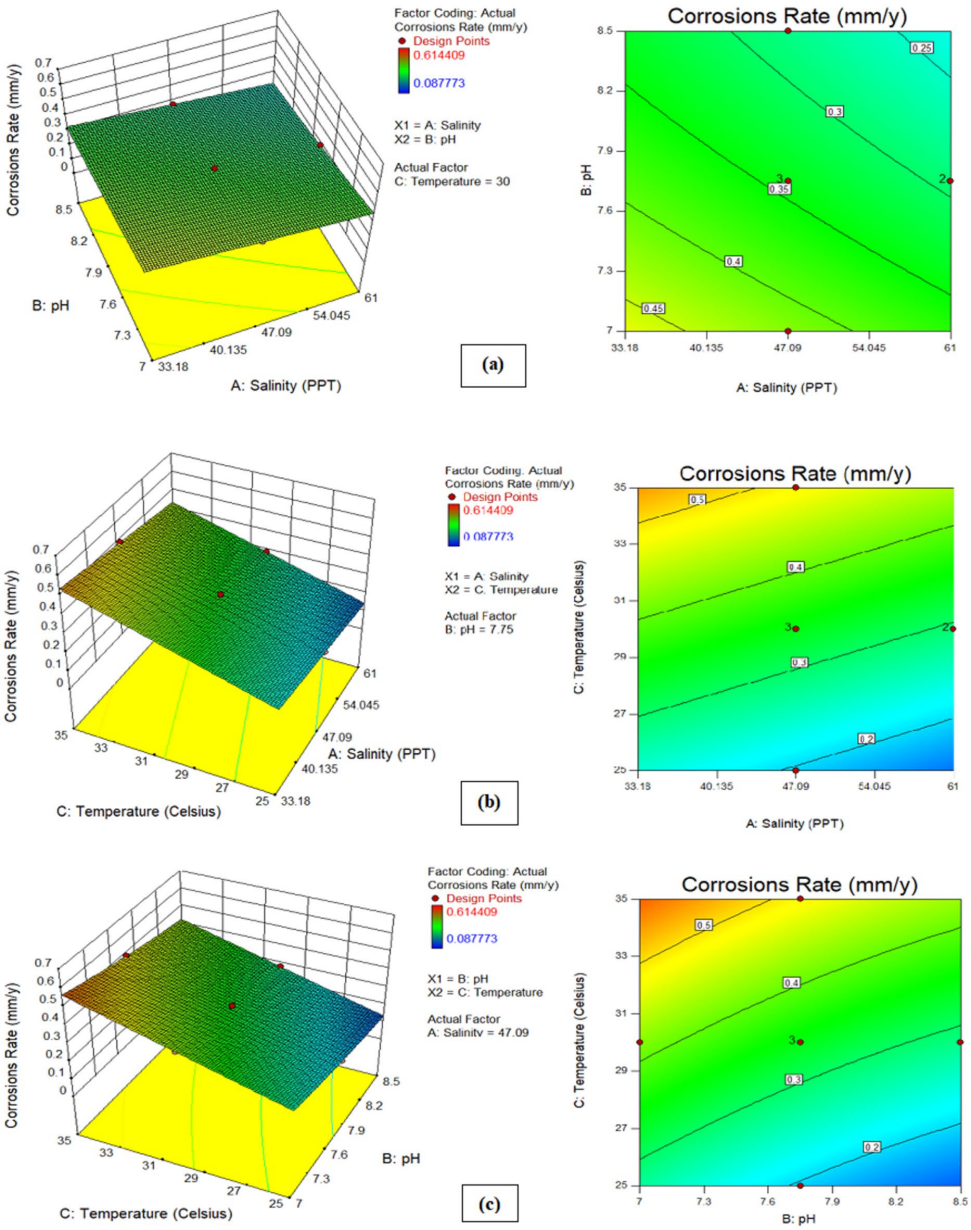
3D and 2D plots of (**a**) pH and salinity, (**b**) temperature and salinity, (c) temperature and pH for corrosion rate.

**Figure 11:**
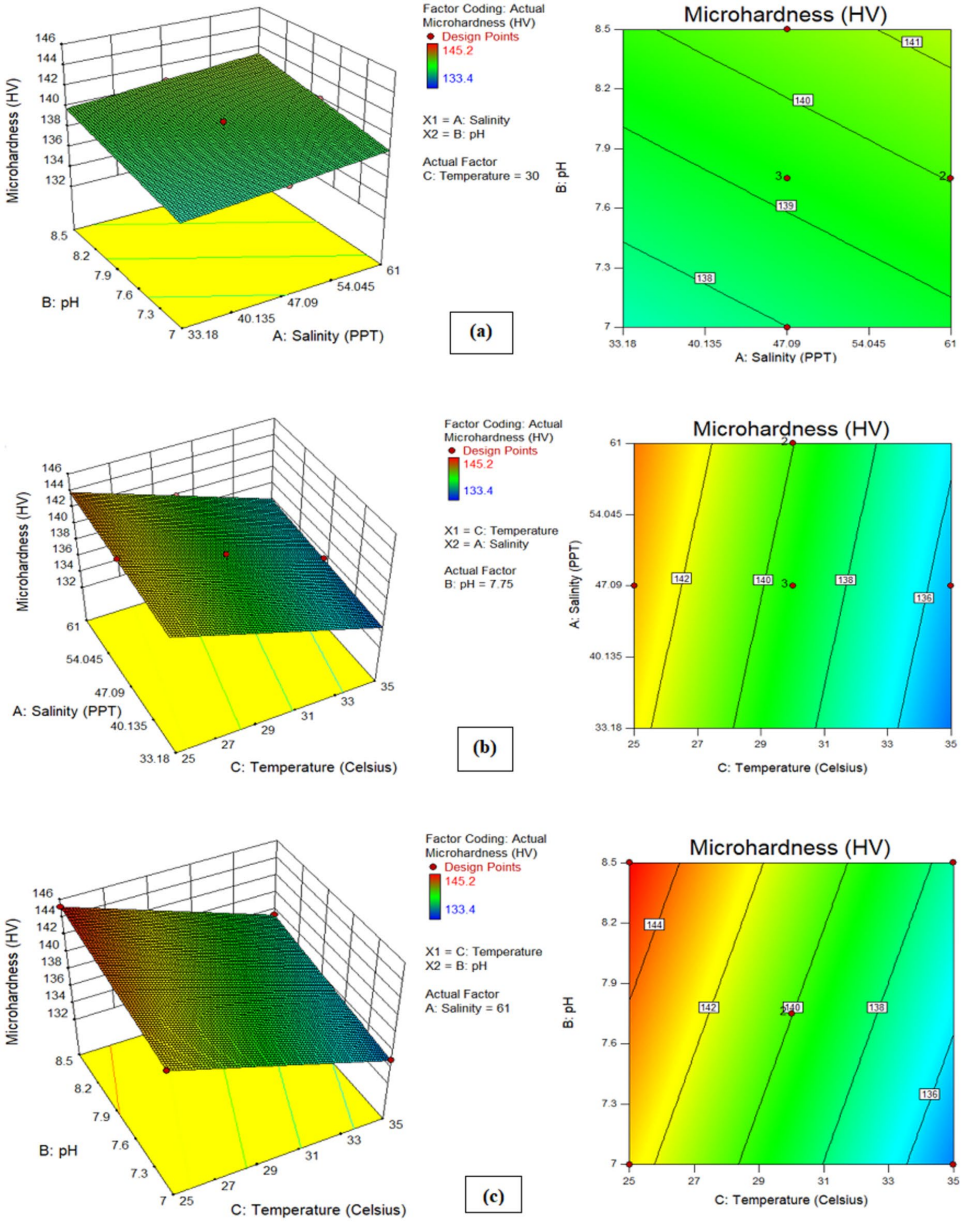
3D and 2D plots of (**a**) pH and salinity, (**b**) temperature and salinity, (c) temperature and pH for microhardness.

**Figure 12:**
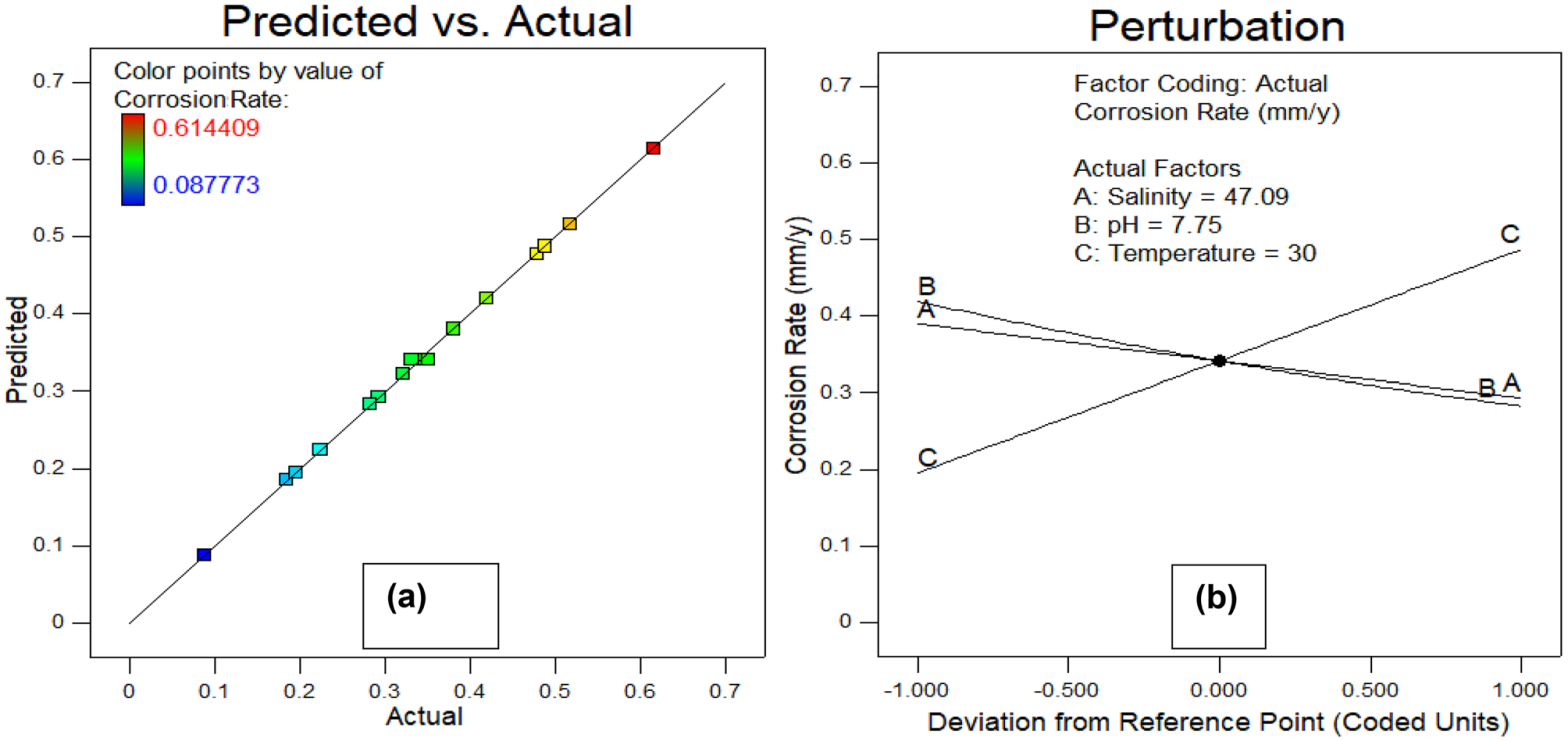
Actual vs predicted (**a**) and perturbation (**b**) plots for corrosion rate.

**Figure 13:**
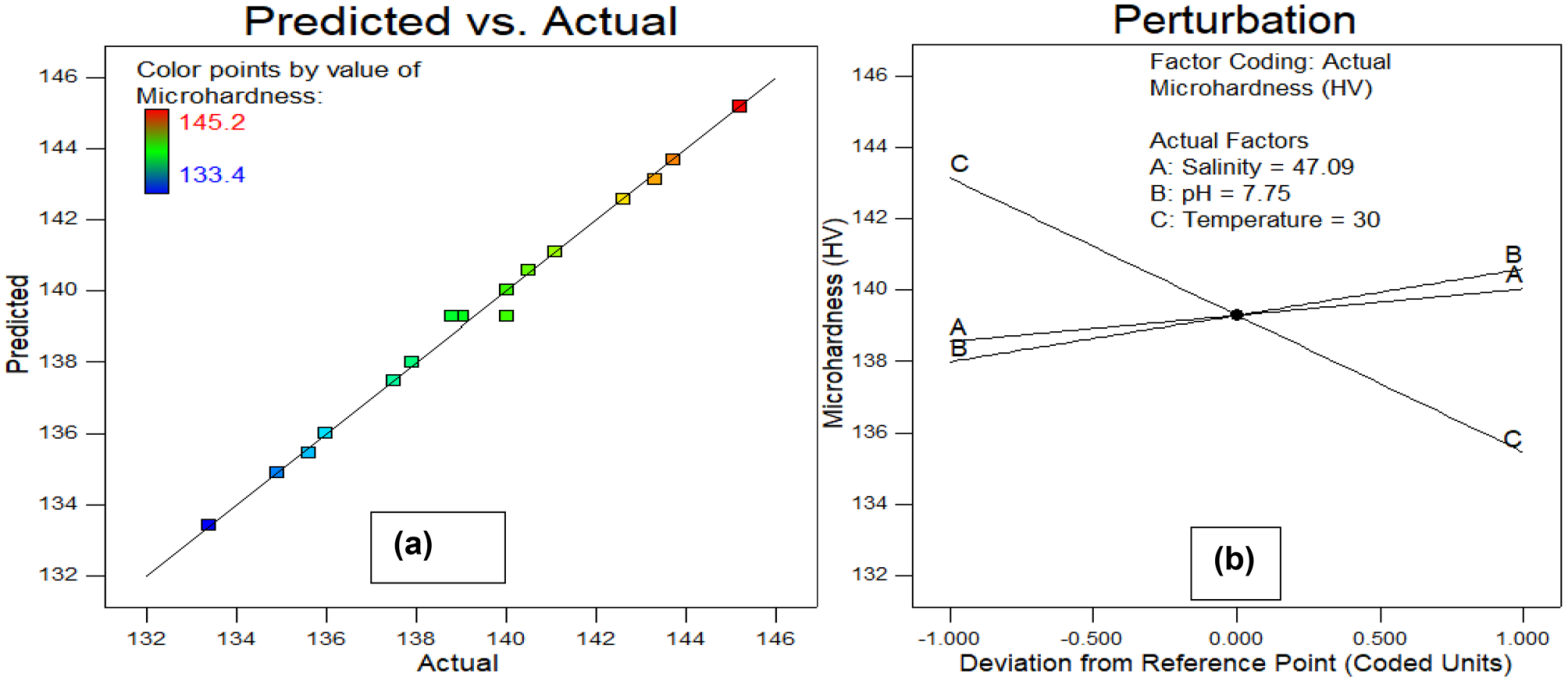
Actual vs predicted (**a**) and perturbation (**b**) plots for microhardness.

To enhance comprehension, it was decided to shed light on the effect of seawater environmental conditions (salinity, temperature, and pH) on both responses. Figure 10 illustrates the correlation between corrosion rate and environmental factors, while Fig. 11 depicts the relationship between microhardness and these parameters. In the experimental scope, each plot showcases how two factors influence one another, while the remaining parameter remains at its central value. The response surfaces in these figures offer a clearer insight into how each factor influences corrosion rate and microhardness. It is observed from Fig. 10a (3D and 2D response surfaces) that pH of seawater has more influence on corrosion rate than salinity. where, as shown in Fig. 10b,c, temperature is found to be the most prominent environmental factor that affects the corrosion rate. These findings are consistent with previous literature^20,46^. Furthermore, Fig. 11a depicts the pH of seawater contributes more than the salinity of seawater to the microhardness response of carbon steel pipe. Subsequently, Fig. 11b,c show that temperature is the most prominent parameter that contributes to the microhardness of carbon steel pipe after 28 days of exposure duration in an artificial marine environment. Figures 12a and 13a show graphs comparing experimental results to expected outputs for both responses. These pictures show a significant connection between real and predicted values, showing a good degree of alignment. Notably, both models exhibit smooth continuity in their variance with no unexpected deviations^47^. The fact that actual data points are close to projected ones demonstrates a great agreement, demonstrating that the quadratic model for corrosion rate response and the linear model for microhardness response are effective in predicting results based on distinct variables^48^. Perturbation graphs serve as crucial diagrams to visualize the impact of all factors within a specific point in the design space. Figures 12b and 13b present plots demonstrating the perturbation of the three factors on corrosion rate and microhardness, respectively. All factors are systematically varied across their respective ranges to depict these responses. Factors A (salinity), B (pH), and C (temperature) were observed within ranges of 33.18 to 61.0 PPT, 7 to 8.5, and 25–35 °C, respectively, allowing us to understand their individual influences on the outcomes. It can be observed from Fig. 12b that the corrosion rate increases from initial range of temperature to final range of temperature whereas slight decrease in corrosion rate was observed with increase in pH and salinity. From Fig. 12b and 13b it is observed that the parameter C(temperature) has greatest influence on both responses (corrosion rate and microhardness) than other A(salinity) and B(pH).

### Finding optimal values of environment parameters (salinity, pH, and temperature) for both responses (corrosion rate and microhardness)

**Figure 14:**
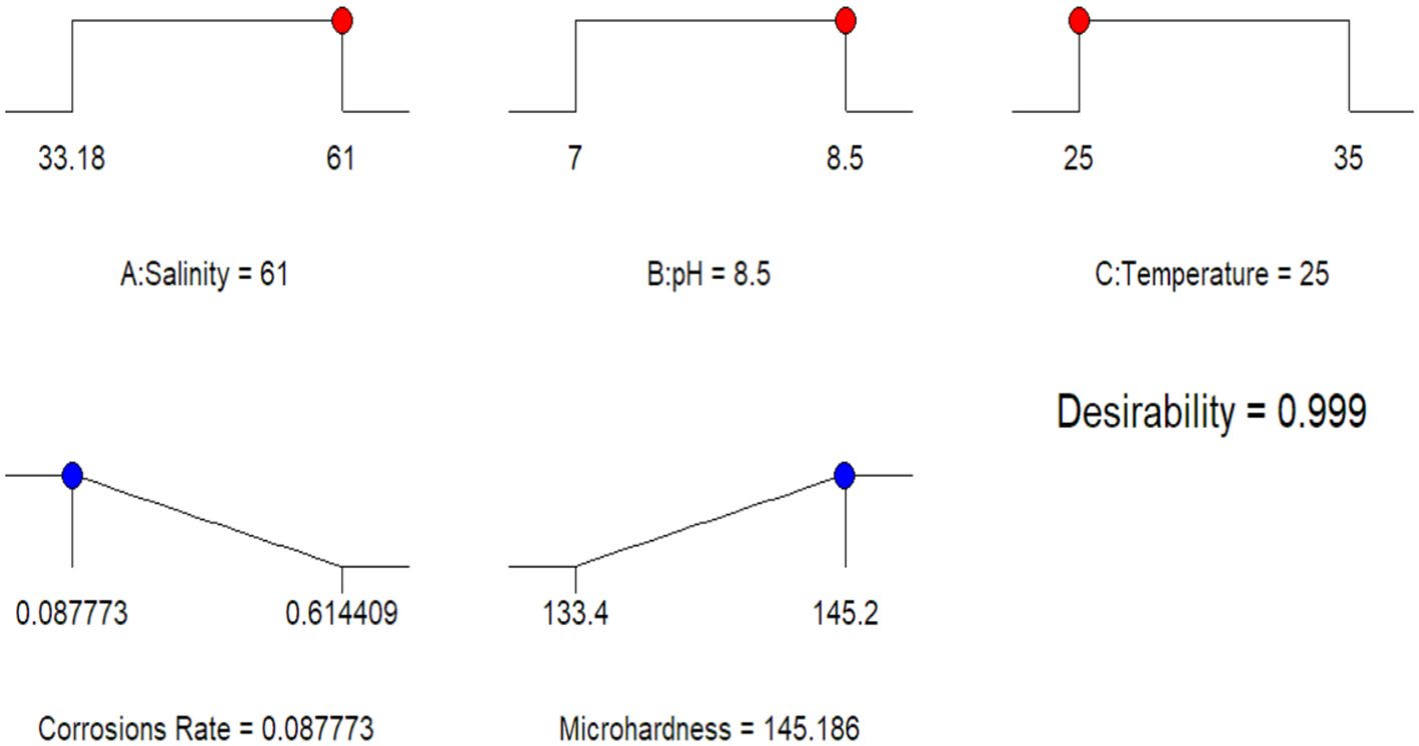
Solution ramp for optimal values.

**Figure 15:**
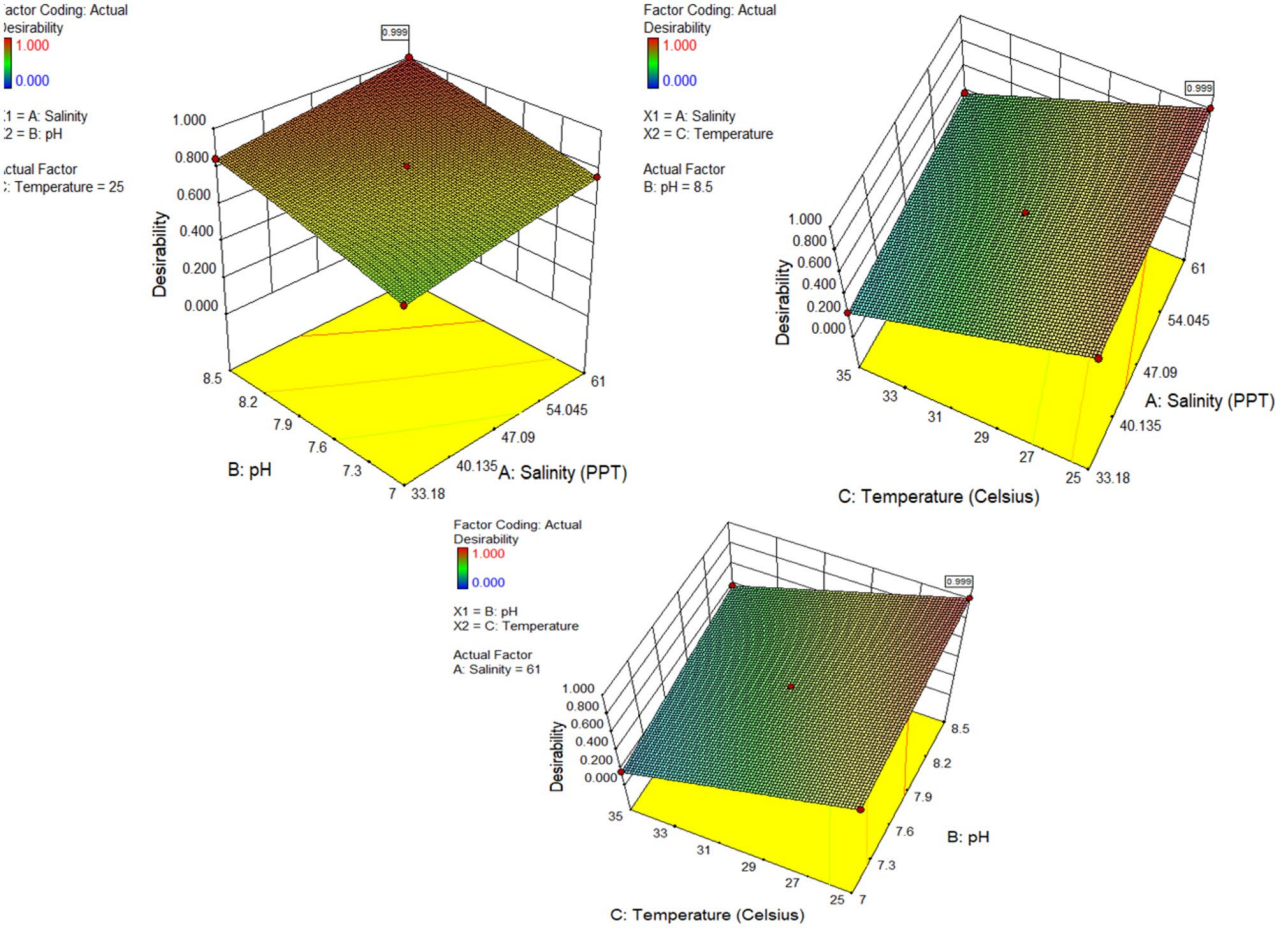
Desirability plots of optimal values related to parameters.

The numerical optimization component of the design expert software predicts the ideal conditions for the environment parameter. The various input constraints and goals of environment parameters are chosen to optimize the response, as presented in Table 10. Solving the prediction equations of the quadratic model and the linear model using experimental results and a surface plot analysis of the responses yielded the best conditions for the input variables. The set of input parameters and the response with the achieved optimum values are also presented in Table 10, where the respective desirability was 0.999. The desirability ranges from 0 to 1^49–52^, depending on the proximity of the response to the target^53,54^. Figures 14 and 15 illustrate the ramps of the optimum model parameters and the 3D desirability (D) generated from multi-objective optimization. The best solution is found in the ramps that show up at 61.0 ppt of salinity, 8.5 of pH, and 25 °C of temperature. The expected results are a corrosion rate of 0.087773 mm/year and a microhardness of 145.186 HV, with a rate of 0.999 for desirability. The multivariate outcome optimization method can be used to find the best seawater environment for CS pipe with the set of parameters and outputs needed to reach the goals. The above-mentioned seawater environmental parameters may be realized with the best corrosion rate and microhardness values at the defined parameters of the multivariate outcome optimization.

**Table 10:** Constraints and goals for optimal response.

Factors	Input factors			Responses (output factors)	
	Salinity (PPT)	pH	Temperature (Celsius)	Corrosions rate (mm/y)	Microhardness (HV)
Value					
Minimum	33.18	7	25	0.087	133.4
Maximum	61	8.50	35	0.614	145.2
Goal	Range	Range	Range	Minimize	Maximize
Optimization Results	61	8.50	25	0.087	145.186
Desirability				0.999 (99.90%)

### Experimental validation of predicted models

Four additional experiments were conducted under identical conditions to those of solutions 1 and 2, aimed at validating the recommendations proposed by the response surface methodology for optimal seawater parameters. Table 11 presents the mean approach employed to study each test factor along with its corresponding absolute relative deviation (ARD) from the anticipated results. The observed ARD was calculated using Eq. (4), allowing for an evaluation of the precision of the predicted model for each parameter^55,56^. The results outlined in Table 11 demonstrate a high level of conformity between the experimental and predicted outcomes, affirming the reliability and predictability of the model, largely owing to minimal variance.

**Table 11:** Experimental validation.

Response	Experimental results	Predicted results	Percentage error (%)
Corrosion rate mm/year	0.08376	0.0877	4.65
Microhardness	143.2	145.186	1.36

4 $$ARD=\frac{Experimental \; results-predicted \; results}{Experimental \; results }\times 100$$

## Conclusion

This study investigated the critical seawater parameters that influence the external corrosion and microhardness of offshore oil and gas ASTM106 grade B carbon steel pipes in various artificial solutions. The immersion test was conducted for 28 days. The FESEM test was used to study the corrosion morphology, and a Vickers microhardness tester was used for microhardness analysis. Furthermore, RSM modelling was employed to find out optimal ranges of seawater parameters like salinity, pH, and temperature. The following conclusion may be drawn from this research work:

- An increase in seawater salinity from 33.18 to 61.10 ppt can reduce offshore pipelines' external corrosion rate by 28%.
- A reduction in seawater pH from 8.50 to 7 results in a 42.54% increase in offshore pipelines' external corrosion rate.
- Seawater temperature is the most prominent parameter, with a mere 10 °C increase in temperature resulting in a 76.13% increase in the external corrosion rate of offshore pipelines and around a 10.99% reduction in the microhardness of offshore pipelines. That is three to four times greater than pH and salinity.
- The FESEM study revealed that large pits formed on the surface of the samples immersed in solution at 35 °C.
- According to RSM modelling, offshore pipelines perform best at salinity, pH, and temperature levels of 61.0 ppt, 8.5, and 25 °C, respectively, with a 0.99 desirability factor.
- The experimental validation for predicted values showed best agreement with 4.65% error for corrosion rate and 1.36 error for microhardness.

## Data Availability

The datasets used and/or analyzed during the current study are available from the corresponding author upon reasonable request.
